# Climatological Aspects of Aerosol Physical Characteristics in Tunisia Deduced from Sun Photometric Measurements

**DOI:** 10.1100/2012/585084

**Published:** 2012-05-02

**Authors:** Mabrouk Chaâbane, Chafai Azri, Khaled Medhioub

**Affiliations:** ^1^Faculté des Sciences, Université de Sfax, B.P. 1171, Sfax 3000, Tunisia; ^2^Institut Préparatoire aux Etudes d'Ingénieurs de Sfax, Université de Sfax, BP 805, Sfax 3018, Tunisia

## Abstract

Atmospheric and climatic data measured at Thala site (Tunisia) for a long-time period (1977–2001) are used to analyse the monthly, seasonal, and annual variations of the aerosol optical depth at 1 **μ**m wavelength. We have shown that aerosol and microphysical properties and the dominating aerosol types depend on seasons. A comparison of the seasonal cycle of aerosol optical characteristics at Thala site showed that the contribution of long-range transported particles is expected to be larger in summer as a consequence of the weather stability typical of this season. Also, the winter decrease in atmospheric turbidity may result from increases in relative humidity and decreases in temperature, leading to increased particle size and mass and increased fall and deposition velocities. The spring and autumn weather patterns usually carry fine dust and sand particles for the desert area to Thala region. The annual behaviour of the aerosol optical depth recorded a period of stead increase started in 1986 until 2001. Trends in atmospheric turbidity after 1988 could be explained other ways by the contribution of the eruption of Mount Pinatubo in 1991 and by local or regional changes in climate or in aerosol emissions.

## 1. Introduction

Atmospheric aerosols play a key role in the functioning of the earth system. Their influence on radiation passing through the atmosphere cannot be neglected, especially in urban or industrialized areas. About three billion tons of particles are injected annually into the atmosphere by natural processes (soil erosion, volcanoes, ocean spray) or by human activities (industrial, traffic). Typical residence time of these particles is around a week in the troposphere. During this period, they absorb or scatter a portion of the telluric and solar radiation (direct effect), they are involved in cloud formation and influence their lifetime and their optical properties (indirect effect). For these two effects, aerosols significantly affect the radiation balance on earth. Because of their small size, these particles are subjected to a long-range atmospheric transport (several thousand kilometres). This ability to transport is such that for some ecosystems and for some elements, aerosols are the major driver of biogeochemical cycling.

To receive comprehensive information about the atmospheric aerosols' optical and microphysical properties, ground-based stations joined together into globally distributed networks and equipped with radiometric instrumentation are now paid special attention. Aerosol optical depth (AOD) data provide a unique opportunity for a comprehensive analysis of diurnal and daily aerosol variations according to aerosol types and landscape.

One of the major components of the aerosol loading is dust which is found over the Pacific ocean and the Mediterranean sea with highest concentrations found over the equatorial and tropical north Atlantic [1]. Mineral dust lifted from arid regions of our planet exerts a large influence on radiative transfer processes [2]. Model studies suggest that the direct radiative forcing of dust on regional as well as on a global scale may be comparable to or even exceed the forcing by anthropogenic aerosols [3, 4]. Nevertheless, net radiative forcing and climate impact of dust particles are still largely uncertain. This fact is due to a still limited knowledge of production sources, transport patterns, particle properties, and evolution and changes during the particles' lifetime.

On a global scale, the dominant sources of mineral dust are all located in the Northern Hemisphere, mainly in North Africa, the Middle East, Central Asia, and the Indian subcontinent [5]. North-West Africa is the most important source of mineral aerosols over the Mediterranean basin [1, 6, 7].

Aerosol and water vapour are somewhat interrelated because aerosols serve as condensation nuclei in the formation of water droplets from water vapour. Next to clouds, aerosols and water vapour are quantitatively the two main parameters affecting transmission of solar radiation through the atmosphere but which are also the least well known. This is mainly due to their variability with location and time and difficulties associated with their measurement.

Attenuation of solar irradiance is strongly dependent on conditions of the sky, cleanliness of the atmosphere, and composition of gaseous constituents. In a clean and dry atmospheric condition, solar irradiance is attenuated by permanent atmospheric constituents of air molecules, gases and ozone, whose contents are nearly invariable. Two additional attenuation processes, which are the absorption by water vapor and scattering by aerosol particles, take place in a real atmosphere. The additional attenuation caused by these two processes is known as being due to the turbidity of the atmosphere.

Atmospheric turbidity is an important parameter for assessing the air pollution in local areas, as well as being the main parameter controlling the attenuation of solar radiation reaching the earth's surface under cloudless sky conditions. Among the different turbidity indices, the Angstrom turbidity coefficient *β* is frequently used [8].

The establishment of the Aerosol Robotic Network (AERONET) which is a federated international network funded in 1993 and coordinated by the NASA that maintains more than 200 permanent automatic sun/sky radiometers worldwide [9, 10] has significantly contributed in the last years to obtain a global coverage and good sampling of aerosol properties.

In this study, two series of sun photometer measurements at Thala site (Tunisia) are used to analyse monthly, seasonal, and annual variations in Tunisia of aerosol optical depth AOD at 1 *μ*m wavelength which is known as Angstrom atmospheric turbidity *β*. The first photometric data are provided by WMO (World Meteorological Organisation) for a long-series experiment (1981–1988) and used to characterize dust particles advected from North-West Africa (Background Air Pollution Monitoring Network BAPMoN) [11]. The results are then compared to those found at the same site with AERONET photometer instrument operated during the period of March–October 2001 by the EMAGPOT Franco-Tunisian project [12–14] and installed by L.O.A (Laboratoire d'Optique Atmosphérique, Lille, France).

Monthly and annual variations of the mean AOD are investigated by examining the origin of the air masses. The temporal extent of Thala region's historical climate record provided the opportunity to examine both seasonal and long-term variability in turbidity in that region. A statistical study of climate data allowed investigating and analyzing the correlation of the atmospheric turbidity with measured meteorological parameters.

## 2. Measured Data Set

### 2.1. Optical Properties of Atmospheric Aerosols

Mie theory characterizes the optical properties of a particle similar in size to the wavelength of the incident light. The theory defines the parameter  *α*_Mie_ = 2*πr*/*λ* where *r* is the radius of the particle and *λ* is the wavelength of the incident radiation. The aerosol properties also depend on their refractive index *m*, which expresses the radiative effect of aerosols according to the nature of the components. Incident radiation on a particle can be either scattered or absorbed. These two phenomena combined lead to the extinction of the transmitted radiation. The efficiency coefficients of extinction and scattering *Q*_ext_ (*α*_Mie_, *m*) and *Q*_scat_ (*α*_Mie_, *m*) define, respectively, the variation of extinction and the scattering in function of the Mie parameter and the refraction index. The additivity of optical properties of spherical particles contained in the atmospheric column allows easily defining the characteristic quantities of particles in the atmosphere. The aerosol optical depth due to extinction  *τ*_aer_^ext^(*λ*, *m*) describes the extinction of direct solar radiation by its scattering and absorption by aerosols. This value is directly proportional to the amount of aerosols and depends on the efficiency factor of extinction *Q*_ext_:

(1) $$\tau_{\text{aer}}^{\text{ext}}\left(\lambda ,m\right)=\overset{r_{\max}}{\underset{r_{\min}}{\int }}\pi r^{2}\cdot Q_{\text{ext}}\left(\alpha_{\text{Mie}},\;m\right)\cdot n\left(r\right)dr,$$

where *n*(*r*) is the aerosol size distribution.

The spectral dependence of extinction is expressed for two the wavelengths *λ*_1_ and *λ*_2_ by the Angstrom coefficient *α*:

(2) $$\alpha =-\frac{\ln\tau_{\text{aer}}^{\text{ext}}\left(\lambda_{1},m\right)/\tau_{\text{aer}}^{\text{ext}}\left(\lambda_{2},m\right)}{\mathrm{In}\lambda_{1}/\lambda_{2}}.$$

This coefficient is an indicator of particle size. It is often used as an indicator of the proportion between the number of large and small particles. The *α* fluctuations reflect changes in size distributions of the aerosol. The Angstrom coefficient increases when particle size decreases, the maximum value of *α* equal to 4 corresponds to molecules. For Saharan dust, this coefficient is low and even negative.

### 2.2. Photometric Measurements from the Ground

The measurement of the attenuation of solar radiation by the atmosphere follows a simple observation technique under the direct sun. The analytical expression is written

(3) $$I\left(\lambda \right)=\;I_{0}\left(\lambda \right)\cdot \exp\left(-m_{\text{air}}\cdot \tau_{\text{tot}}^{\text{ext}}\left(\lambda \right)\right).$$

The irradiance *I*_0_(*λ*) is the measurement of solar radiation outside the atmosphere,  *I*(*λ*) is the radiation attenuated by the atmospheric components, and *m*_air_ is the air mass which could be approximated by the relationship:

(4) $$m_{\text{air}}=\frac{1}{\cos\theta_{s}},$$

for solar angles less than 75°.

The total optical depth *τ*_tot_^ext^(*λ*) is composed of two terms outside the absorption bands of gaseous. One is due to molecular scattering *τ*_Ray_^dif^(*λ*)  and the other is due to the scattering and absorption by aerosols  *τ*_aer_^ext^(*λ*):

(5) $$\tau_{\text{tot}}^{\text{ext}}\left(\lambda \right)=\tau_{\text{aer}}^{\text{ext}}\left(\lambda \right)+\tau_{\text{Ray}}^{\text{scat}}\left(\lambda \right).$$

Under these conditions, the aerosol optical depth determined from photometric measurements is given by

(6) $$\tau_{\text{aer}}^{\text{ext}}\left(\lambda \right)=\frac{1}{m_{\text{air}}}\cdot \ln\left[\frac{I_{0}\left(\lambda \right)}{I\left(\lambda \right)}\right]-\tau_{\text{Ray}}^{\text{scat}}\left(\lambda \right).$$

The measured optical depth includes all aerosols in the atmospheric column, but it is usually largely due to tropospheric aerosols concentrated in the lower layers. This photometric method can be considered the reference technique for measuring the aerosol optical depth in the solar spectrum. Photometric measurements are used to determine the atmospheric turbidity factor *β* and Angstrom coefficient *α* [15] from data of aerosol optical depth. These coefficients are related by the Angstrom formula:

(7) $$\tau_{\text{aer}}^{\text{ext}}\left(\lambda \right)=\beta \cdot \lambda^{-\alpha }.$$

*β* expresses the amount of aerosols in the atmosphere and characterizes the degree of air pollution. *α* is an indicator of the size of atmospheric particles. 

The experimental determination of the Angstrom's turbidity coefficient *β* requires measurements of spectral direct normal irradiance at two wavelengths where absorption is negligible. The method consists in making a direct fit of Angstrom equation (7) to the experimental data. This fit produces a single value for each of the coefficients *α* and *β* valid for the whole band. Values of the Angstrom turbidity coefficient have been determined using spectral sun photometer data.

Data reported in this study are related to two different periods. The first spreads from 1981 to 1988 and measurements were made with a sun-sky radiometer installed at the meteorological Thala station and operated by WMO (World Meteorological Organization) in the framework of the global atmospheric background monitoring for selected environmental parameters (WMO, BAPMoN data for atmospheric aerosol depth) held on several sites on the globe at a rate of three measurements per day [16]. Volz introduced a hand-held Sun photometer 50 years ago [17]. Improved versions of this instrument [18] were used in various networks, including 95 stations of BAPMoN.

A Sun photometer should detect a relatively narrow band of optical wavelengths since the optical depth of the clear sky is strongly wavelength dependent. Optical interference filters have long been used to meet this requirement, but such filters are expensive and subject to unpredictable and very significant degradation. Filter degradation is such a serious problem that it caused the international BAPMoN network to be closed.

The second period is only for 2001 and which data are provided by CIMEL sun/sky radiometer within AERONET. This is an automatic, robotically operated instrument. Two detectors are used for the measurements of direct sun and sky radiance. Spectral observations of sun radiance are generally made at seven spectral channels: 340, 380, 440, 500, 675, 870, and 1020 nm, while measurements of sky radiance are made at 440, 675, 870, and 1020 nm every 15 minutes [9, 10]. A flexible inversion algorithm developed by Dubovik et al. [19] is used to retrieve columnar aerosol volume size distribution, real and imaginary refractive indices, and single scattering albedo from direct sun and diffuse sky radiance measurements.

It is generally accepted that the calibration is the paramount problem of sun photometry. The experience shows that good primary calibrations are only possible from high-altitude stations with exceptionally clear sky conditions. Experiments from rockets demonstrate the validity of the high mountain calibrations by the Langley method but only if the calibration days are carefully selected [20].

## 3. Climatic Characteristics of the Measurement Site

Thala station occupies a strategic position in point of view of meteorology (the only mountain resort in Tunisia, at an altitude of around 1090 m). It was chosen in nearly the majority of projects using the national meteorological network. It was place of a draft measure of pollution (chemical precipitation, aerosols and gases) in the late seventies with equipment provided by the World Meteorological Organization (sampling of rainfall using an automatic rain gauge). Thala measurement site (35° 33′ North; 08° 41′ East; 1091 m asl) is located in the mountains, the vegetation consists mainly of wild plants such as cactus. Olives are also cultivated but rarely. The lands around the station are operated as a breeding ground.

Since its opening, the meteorological station operates with conventional measuring instruments normed by WMO. The measured data are daily or monthly submitted on technical documents to regional center of the subdivision of Sfax where they will be corrected and then sent to the national center of Tunis to capture. They are stored in a database after undergoing digital control operations (application of several statistical procedures on data sets, error identification and correction or elimination). The dataset presented in this section is a result of a statistical analysis to a measurement series between 1977 and 2001.

**Figure 1:**
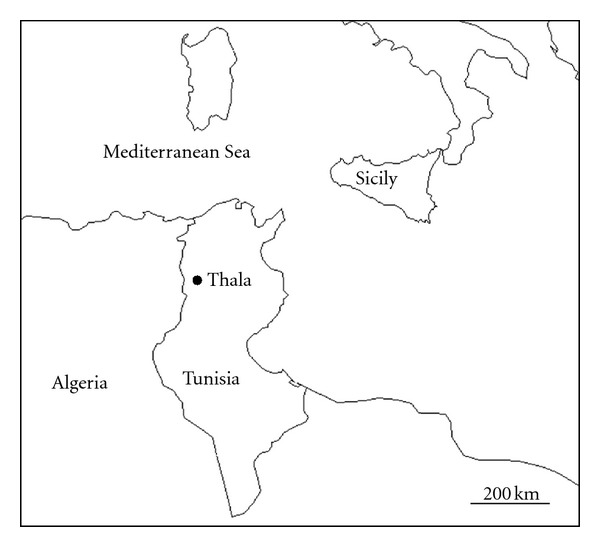
Geographical location of Thala Tunisian site.

Figure 1 shows the geographical location of the site selected for this study. Thala is a small town situated in the Middle West of the Tunisian territory at about 250 km south of the capital of Tunisia and close to the Algerian border. The measurement site is located in the meteorological station (about 6 km from Thala city) which is a part of the World Meteorological Organization network. Thanks to its geographical position, the instrument is particularly appropriate to detect both frequent dust outbreaks from the African Sahara and marine particles from the Mediterranean Sea. A minor impact of anthropogenic pollution is expected at this site.

### 3.1. Air Temperature Variations

**Figure 2:**
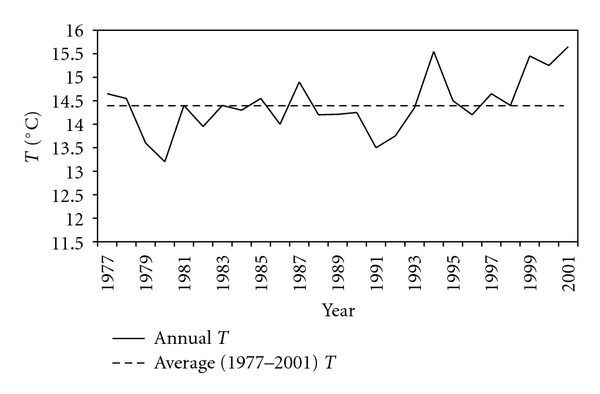
Annual averaged temperature at Thala site between 1997 and 2001.

Thala is the coldest region of Tunisia. Mean monthly values of temperature vary between 5.7°C in January and 24.6°C in July (Table 1). Annual averaged temperature is 14.4°C computed for the time period 1977–2001. It varies between 13.2°C in 1980 and 15.7°C in 2001. Apart a notable peak recorded in 1994, the period spanning from 1977 to 1995, has been characterized by mean temperatures generally below the annual average. However, we note between 1996 and 2001 a considerable growth exceeding 1°C at the annual scale (Figure 2).

At the seasonal scale, we note that winter in Thala is characterized by low temperature with mean value of 6.5°C. The coldest winter from the studied period has been observed in 1981 with averaged value around 5°C and a number of 28 days where temperature is less than 0°C. Spring and autumn are characterized by temperature varying between 12.1°C and 15.7°C. The highest seasonal temperatures for spring and autumn are, respectively, 14.6°C (1981) and 17.2°C (1978) and the lowest are 9.5°C (1980) and 13.9°C (1979).

The diurnal thermal amplitude (difference between maximum and minimum temperatures) varies from one month to another. It peaked in summer months, with a value equal to 11.2°C. The minimum is observed in January and December, with a value not exceeding 4.2°C.

### 3.2. Wind Speed and Direction

Although Thala site is located at altitude (1091 meters), we find that the wind is relatively moderate to fairly strong, as showed by the weakness of calm winds with an average frequency of 1.8% and strong winds with a frequency not exceeding 1.9%. We note also that at annual basis, half of the wind speeds did not exceed 5 m/s.

 On the other hand, the wind blows from all directions and this in proportions is quite different in both frequencies and speeds. Winds from NNW, N, and NW sectors are still the most frequent and also the strongest. We can conclude that the prevailing wind at Thala station is the North to North-West, with a total frequency exceeding 47%. Wind sectors ESE, E, and SE are rare and blow with relatively low speeds (Table 2). South blowing winds are relatively frequent in the period between April and October.

Moreover, we note that the most common values of wind speed are those between 3 and 8 m/s (Table 3). Strong winds with average frequency of not exceeding 1.9% are rare. They blow from NNW direction, South and North, with frequencies equal, respectively, to 27, 14, and 13%. It is noteworthy that despite the low frequency of winds from a southerly direction (only 9.3% of total sales), the majority of these winds are strong (14% of the winds).

Considering the geographical position of Thala station, the sand storm is a meteorological phenomenon very uncommon. During the period 1977–2001, this phenomenon has shown only in 1977, 1978, 1998, and 2001.

The sirocco, known in Tunisia as the “Chehili”, is a wind of dynamic origin, blowing from South and Southwest areas. It is usually accompanied by a very significant rise in temperature and a remarkable drop in relative humidity. A total of 263 days of sirocco are registered at Thala station with the majority during the summer season (June, July and August) (Table 4). This shows that our station, despite being a mountainous region near the north-west, is not sheltered from the sirocco blows.

### 3.3. Relative Humidity

**Figure 3:**
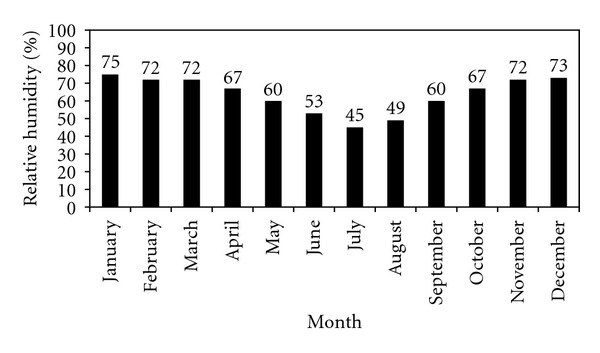
Monthly means of relative humidity at Thala station.

Thala station has a fairly high relative humidity during the months of September to May. It ranges from 75% in January and 60% in May and September. It suffered a significant decline during the three summer months, due to the continental aspect of the station (only 50%). In the beginning of the autumn, it climbs rapidly above 60% (Figure 3). A wide daily variation of humidity is much more significant. Maximum values are observed between midnight and early morning hours, then declines rapidly to reach the minimum around 15 h.

### 3.4. Amount of Rainfall

**Figure 4:**
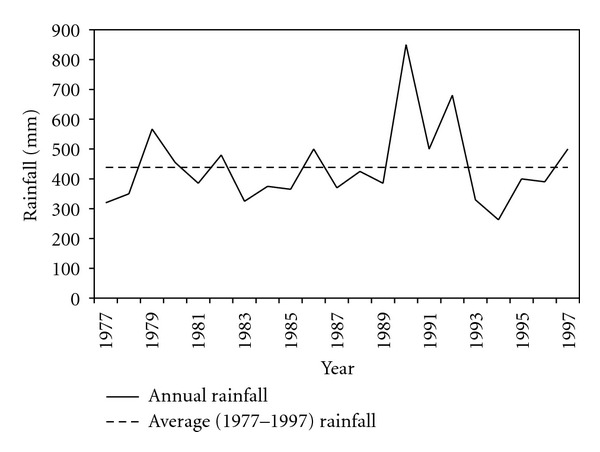
Annual rainfall in Thala station for (1977–1997) period.

With an annual rainfall averaged over the period 1977–1997 equal to 440 mm, the Thala station belongs to a region ranked as moderated rainfall in Tunisia. Between 1981 and 1988 annual amount of rain varies between around 330 and 500 mm (Figure 4). In 2001, the recorded annual value falls to 313 mm.

## 4. Monthly and Seasonal Variations of Aerosol Optical Properties

### 4.1. Aerosol Optical Depth AOD

The mean monthly values of AOD measured for the two studied periods are deduced from individual measurements performed at Thala site with sun photometer instrument. The number of observations for each month is illustrated in Table 5.

**Figure 5:**
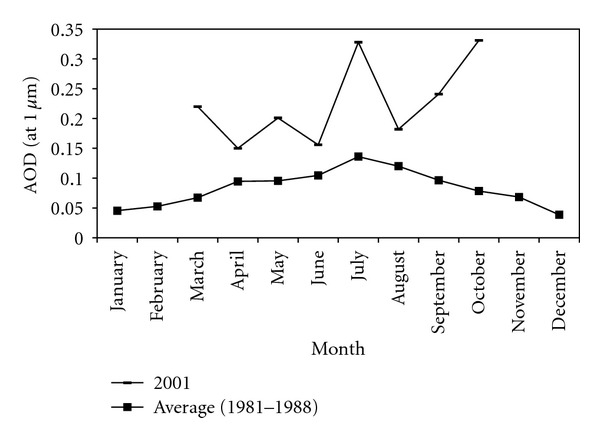
Mean monthly variations of AOD (*λ* = 1 *μ*m) at Thala site for the two series of measurements.

The AOD (*λ* = 1 *μ*m) monthly variability is clearly revealed by Figure 5 showing different results for the two studied periods. First, for the period (Mars–October 2001), data are determined by high monthly variability which will be explained latter, with two notable peaks in July and October. In the contrary, the second period between 1981 and 1988 is characterized by small monthly variability showing that AOD (*λ* = 1 *μ*m) takes averaged values larger than 0.1 from June to August (summer) and lower or equal to 0.1 from September to May with a peak in July and a minimum in December to January. These results are quite comparable to those found for 1999 at Sidi Bou Saïd station (Northern Tunisia) in spite of the difference between the geographical characteristics of the two sites. We showed [21] that mean monthly values of Linke turbidity factor *T*_*L*_ present two levels. The first, with highest turbidity, is observed between April and August. The second, characterized by lowest values, is related to rest of the year (September to March). We note moreover that, Linke's *T*_*L*_ factor refers to the whole solar spectrum and represents the turbidity caused by aerosols and water vapour, which affects solar radiation by absorption in the visible and near infrared regions. On the contrary, *β* coefficient has been accepted as an index of the turbidity caused just by aerosols, as it represents the aerosol load in the air and it is obtained from spectral measurements. 

The larger Angstrom turbidity coefficient values observed in summer months may be due to a larger concentration of aerosol size distribution characterized by higher scattering and/or absorption coefficients. In fact, turbidity depends on the vertical profile of the aerosol extinction coefficient that is made of two parts: the scattering and the absorption component [22]. 

Probably, turbidity seasonal variability revealed by Figure 5 is mainly determined by the seasonal dependence of aerosol removal processes. The weather stability typical of summer regimes favours the accumulation of atmospheric particles over North Africa. As a consequence, the contribution of long-range transported particles is expected to be larger in summer regimes. Moreover, the aerosol wet removal is practically absent over the east Mediterranean basin in summer and the larger amount of solar radiation reaching the earth's surface may favour photochemical reactions that affect optical and microphysical properties of the accumulated atmospheric aerosols. Also, the winter decrease in atmospheric turbidity may result from increases in relative humidity and decreases in temperature (as it is presented in Section 3), leading to increased water uptake in the aerosols, hence increased particle size and mass and increased fall and deposition velocities. The two main causes of the low aerosol optical characteristics in the winter season are washout by rainfall and high relative humidity leading to aerosol size increases and subsequent deposition. 

**Figure 6:**
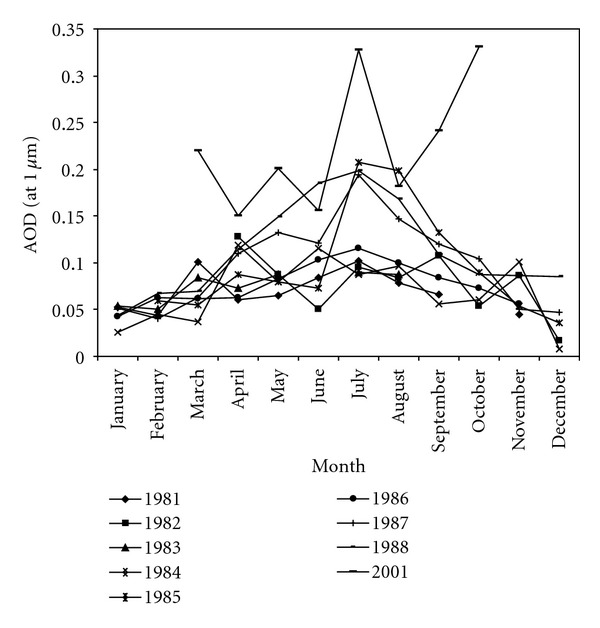
Monthly variations of AOD (*λ* = 1 *μ*m) at Thala site for each studied year.

It is worth observing from Figure 5 that some months from the first period (1981–1988) are characterized by mean AOD (*λ* = 1 *μ*m) values larger than 0.15. Moreover, for 2001 studied period, some averaged monthly values exceed 0.2 with rapid change from month to month. This may indicates long-range transport of aerosols in Thala. The high-aerosol-load days are attributed to Saharan dust. In fact, we have already mentioned (Section 3) that during the two studied periods, sandstorms have only been recorded in 2001. We can also note that the nonhomogeneity between the monthly turbidity trends for the two series of measurements (Figures 5 and 6) could be explained, among others, by climate change effects. This is also well proved by the rise of more than 1°C in annual averaged temperature between 1981 and 2001 and on the other hand by a decrease in rainfall amount in 2001 compared to the first period. 

From Figure 6, we could also observe that particularly for 1987 and 1988, turbidity at Thala presents two levels. The first, with averaged *β* values greater than 0.1, is related to the spring-summer period (from April to September) reaching a maximum of around 0.2 in July. The second period spreads from October to March and for which monthly averaged turbidity is lower than 0.1. 

The spring season (March to May) over Thala is characterized by moderately high temperature and wind speed and also by low relative humidity as shown in Section 3. These spring weather patterns usually carry fine dust and sand particles from the desert area to Thala region. 

The summer season (from June to August) is characterized by high temperature, low relative humidity, and relatively low wind speeds over the studied site. This climate situation leads to increased photochemical processing in the atmosphere and hence greater anthropogenic aerosol production. The aerosols formed through primary emissions and secondary reactions are thus potential causes for the observed turbidity values during this season. In addition, strong vertical temperature gradients in the Thala area in late summer lead to enhanced vertical convection, carrying aerosols aloft and enhancing turbidity. Similar results have been found by Perrone et al. [7] for one year (March 2003 to March 2004) of sun-photometer measurements over South-East Italy. They have shown that aerosol optical and microphysical properties and the dominating aerosol types depend on seasons. 

In conclusion, it is difficult to attribute seasonal trends in turbidity to anyone causal factor. Meteorological parameters are only one of many factors that can influence turbidity at any given location. Other climatic factors include convective activity, seasonal differences in air mass origin, and the synoptic situation, as well as dispersion, transformation, and removal processes. 

These preliminary results that based on a yearly comparison of measurements reveal that concentration, size distribution, and chemical composition of aerosol particles depend on seasons and lead at first of the assumption that the higher aerosol loads are characterized by a predominant contribution of larger, scattering particles over west regions of Tunisia in summer.

### 4.2. Angstrom Coefficient and Volume Size Distribution

Recent studies have shown many useful applications of measures of Angstrom coefficient to characterize the radiative properties of aerosols [23, 24]. These measurements are also used to determine the size distribution of aerosols which is the most important parameter to characterize a population of aerosols. The size distribution of atmospheric aerosols can be described in terms of modes that characterize their size range. The submicron particles are classified into accumulation mode (0.05 < *r* < 0.5 micron), Aitken mode (0.01 < *r* < 0.05 microns), and the nucleation mode (*r* < 0.01 micron). Coarse particles are defined for a range of radius *r* > 1 micron [25]. In the accumulation mode, we found organic compounds and sulfates from industrial emissions of sulfur dioxide (SO_2_). 

The electromagnetic scattering theory indicates that small particles with radius comparable to the wavelength *λ* have large *α* values of order of 1, while large particles have small *α* values close to 0. Typical *α* values that range 0.5~1 for accumulation mode aerosols in the urban type air mass were observed by ground-based measurements. Small *α* values (less than 0.5) originate from areas of prevailing soil-derived particles such the area near the Saharan desert. High *α* values (greater than 1) are caused by anthropogenic aerosols around industrial areas or by biomass burning aerosols [19, 23]. 

**Figure 7:**
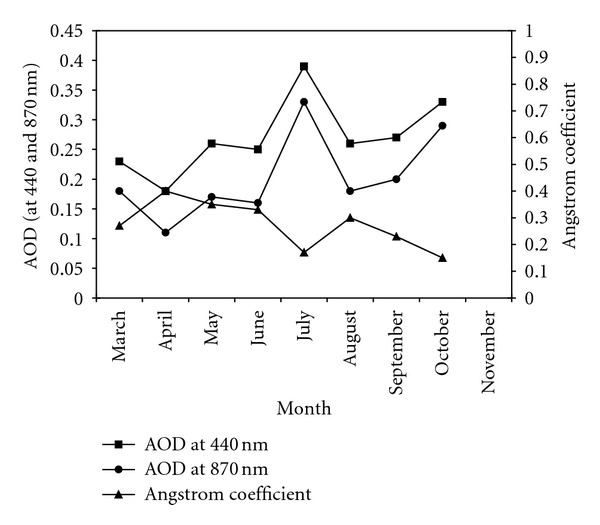
Monthly evolution of mean AOD (at 440 and 870 nm) and Angstrom exponent *α* at Thala site in 2001.

The Angstrom exponent *α* is defined by the spectral dependence of the aerosol optical depth. The temporal evolution of the Angstrom exponent (computed from AOD values at 440 and 870 nm) is also reported in Figure 7. We clearly observe that AODs and Angstrom exponents are following opposite trends at Thala site for 2001 period. 

It is worth observing that July and October 2001 are characterized by mean monthly AOD larger than 0.25 (Figure 7). Relatively low averaged Angstrom coefficient *α* computed from 440 and 870 *μ*m wavelengths (less than 0.2) is also observed. The high turbidity linked to the low values of *α* recorded in these two months suggests that the influence of the southwesterly winds blowing in from the Sahara is maximal [13]. 

**Figure 8:**
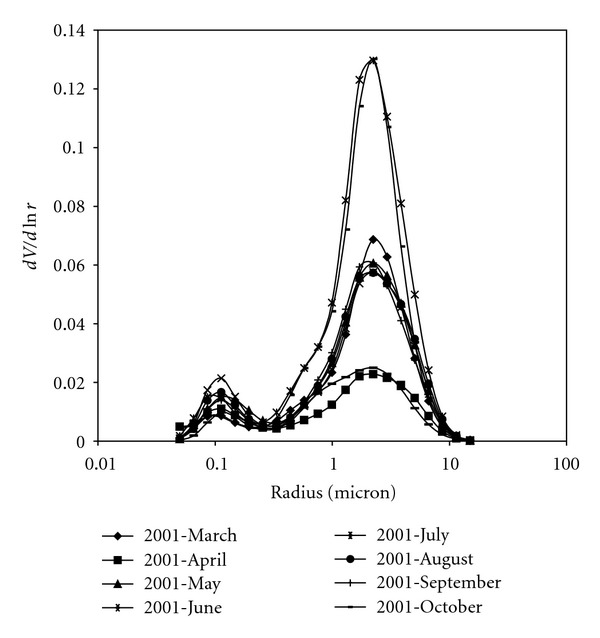
Mean volume size distribution at Thala site for 2001 months.

Figure 8 shows the mean volume size distribution obtained at Thala site by averaging different volume size distribution profiles retrieved during the 2001 months. The number of inversions per month is illustrated on Table 6. The domination of coarse mode particles in desert aerosols is clearly pointed out: particles radius peak values span the 1.7–3 *μ*m range. Aerosols with a dominant contribution of moderately absorbing particles are advected from Europe and West Africa. Air masses from these regions have the common property to travel across large cities and industrialized areas before reaching Thala region and as a consequence these air masses can be affected by urban-industrialized aerosols [6] as well as by seasonal biomass burning [26].

Monthly volume size distributions plotted on Figure 8 reveal that the coarse mode is predominant and as a consequence, Angstrom coefficient *α* takes averaged values lower than 0.5, in accordance with typical desert properties. In fact, AERONET sun photometer measurements performed from 1993 to 2000 at Cape Verde (Africa) during dust outbreaks have shown that the median radius of the coarse mode was 1.9 ± 0.03 *μ*m [27]. Also, the scanning electron microscopy analysis of several dust samples from rainfall residues collected at Leece (Italy) during dust storm occurring from April to June 2002 have provided size distributions with median radius values between 0.85 and 1.2 *μ*m [28]. 

Previous studies [7, 29, 30] have shown that the optical and chemical properties of dust particles are quite less dependent on wavelength. Then, lower AOD dependence on wavelength can be used as marker for the dominant contribution of desert type particles. As a consequence, the Angstrom coefficient *α* takes values ranging from 0.1 to 1 along dust events. The AOD wavelength dependence is determined by size distribution, shape, and chemical composition (real and imaginary refractive indices) of aerosol particles. Characterizations of water insoluble components of Sahara dust samples from rainfall residues have shown that dust particles with a high content of illite are mainly advected over the Mediterranean basin during dust storms [28]. These results are also in accordance with those obtained by Avila et al. [31] by analyzing 11-year records of Africa dust rain in the Montseny Mountain (north eastern Spain). They have observed that illite was the most abundant mineral identified in all dust samples by X-ray diffraction and that the illite concentration was 41% when the source region was western Sahara, and 34.5% for the air masses comes from central Algeria. 

## 5. Interannual Variability of Atmospheric Turbidity 

**Figure 9:**
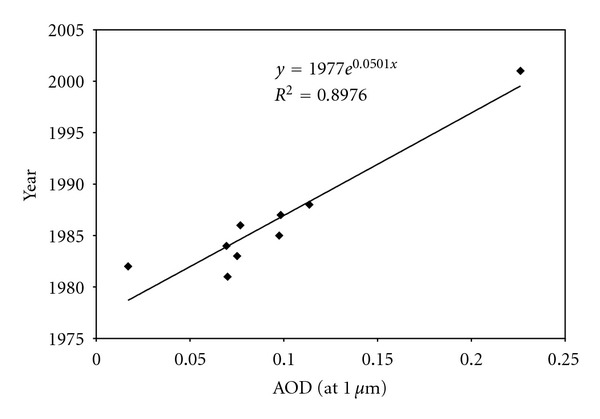
Annual variation of mean AOD (*λ* = 1 *μ*m) at Thala site for the two series of measurements.

In order to examine long-term trends in turbidity over central west Tunisian region, moving averages were computed at Thala station. Although interannual variability in turbidity is evident, the first data series (1981–1988) is generally characterized by stable turbidity. However, a linear model fitted to the Thala time series shows long-term trends in turbidity. Long-term averaged turbidity is around 0.1, *β* has, on average, increased by approximately 0.044 between 1981 and 1988 and by 0.156 between 1981 and 2001.  Figure 9 presents the annual variation of averaged *β* Angstrom turbidity coefficient for the two studied periods: 1981 to 1988 and 2001. Relatively low values (between 0.05 and 0.12) are observed during the first period (1981–1988); a rapid increase appears in 2001 with an averaged annual value around 0.23. A linear increasing trend is observed for the variation of *β* coefficient between 1981 and 2001. Minimum averaged value of the turbidity coefficient (*β* = 0.017) occurred in 1982 was accompanied by low air temperature (averaged yearly temperature near 14°C) and high rainfall (around 480 mm per year) in the period under study. 

**Figure 10:**
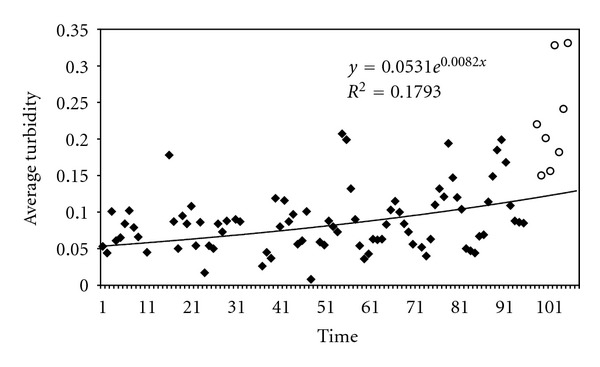
Linear trend fit to monthly AOD (*λ* = 1 *μ*m) at Thala site from January 1981 to December 1988 (series 1) and from March to October 2001: the last eight points (series 2).

In Thala site, a period of steadily increasing turbidity commenced in 1986 and continued until 1988 (Figures 9 and 10) and then to 2001. In the contrary, turbidity is generally stable (0.05 < *β* < 0.1) through the rest time series (1981–1985). Between 1988 and 2001, there are insufficient turbidity data to put in evidence the increase in aerosol load in Thala region and to enable the assessment of any possible effects on the aerosol climatology. However, short-term trends after 1988 can be explained by the contribution of the eruption of Mount Pinatubo. Power et al. [32] have shown a rise in Angstrom turbidity starting in 1991 to 1997 over south African sites (mainly Cape Town, Pretoria and Upington). The authors explain this trend by aerosols ejected from the June 1991 eruption of Mount Pinatubo. Anomalously high aerosol optical depth (AOD) values and low global direct radiation were observed in mid 1991 over Cairo and early 1992 over Aswan [33]. Compared to El-Chichon's eruption, that of Pinatubo was distinguished by a large magnitude, leading to a 50% increase in stratospheric extinction as demonstrated by Michalsky et al. [34]. Some relevant Pinatubo AOD measurements can be also found in Schmid et al. [35]. These aerosols first produced a strong depleting effect on direct radiation, as documented elsewhere but subsequently decayed exponentially over time. The increase of particle load in the atmosphere site may probably result on the possible effects of this fact on the aerosol climatology. In fact, volcanic eruptions eject gas and dust into the earth's upper troposphere, where dispersion processes lead to worldwide transport of the ejected material. Sulfur dioxide gas emitted by the volcanoes is oxidized over a period of weeks to form sulfuric acid gas. This gas nucleates and/or condenses to form aerosols that impact the direct solar radiation. 

Furthermore, the long-term trend in turbidity that does exist in Thala should also be interpreted by local or regional changes in climate or in aerosol emissions (natural or anthropogenic). As with the seasonal trends identified above, meteorological parameters are one of many factors that can influence turbidity at any given location. As is shown in Section 3, 2001 has been characterized by particular meteorological conditions, compared to the first period of measurement (1981–1988): mean annual temperature, rainfall amount, and sandstorm phenomenon.

## 6. Conclusion

This research provides a preliminary climatology of aerosols previously unavailable for Thala Tunisian site. A long-time period of data has been used to analyse the monthly, seasonal and annual variations for the atmospheric Angstrom turbidity coefficient *β* (AOD at *λ* = 1 *μ*m) computed for two different periods (the first: 1981–1988; the second: March–October 2001). It was found that in spring and summer seasons, the aerosol optical characteristics recorded significant high values, due to the effects of sand and dust particles emitted from the African Sahara. In summer, photochemical processes became the main origin of the aerosol and this leads to slight increases in the values of aerosol optical characteristics, despite lower wind speeds relative to other seasons. In the autumn and winter seasons, the lowest aerosol optical characteristics were measured over Thala. This was due to the washout by rain and high relative humidity. 

The interannual variability of the Angstrom turbidity coefficient *β* is also investigated. *β* coefficient recorded a period of steadily increasing turbidity commenced in 1986 until 2001. Short-term trends after 1988 are explained among others, by the contribution of the eruption of Mount Pinatubo. The long-term trend in turbidity is interpreted by local or regional changes in climate or in aerosol emissions. 2001 has been characterized by particular meteorological conditions compared to the first period (1981–1988). 

It is shown that aerosols of different origin and type can be advected at this region. However, depending on travel distance and residence time over source regions and monitoring site, the particle number concentrations, the physical and chemical state, and thus the optical properties of aerosols change. As a consequence, there is no straightforward one-to-one relationship between enhanced aerosol load and long-range transport from Europe and/or Africa.

## Figures and Tables

**Table 1:** Monthly averaged temperature at Thala site.

	January	February	March	April	May	June	July	August	September	October	November	December	Average
Temperature (°C)	5.7	6.6	8.8	11.3	16.3	21.3	24.6	24.4	20.2	16.5	10.3	7.1	14.4

**Table 2:** Monthly frequencies of mean wind directions at Thala site.

Wind direction	January	February	March	April	May	June	July	August	September	October	November	December
N	12	11	15	13	15	17	17	18	16	12	13	12
NNE	2	1	2	1	2	3	3	3	3	2	2	1
NE	1	2	2	2	3	3	3	3	3	2	1	1
ENE	1	1	2	1	2	2	3	3	3	2	1	1
E	1	1	2	1	2	2	2	2	2	2	1	1
ESE	1	1	1	1	1	2	1	1	1	1	1	0
SE	2	2	2	3	4	6	5	5	5	6	3	1
SSE	3	4	4	8	9	11	9	7	6	6	3	1
S	6	5	7	10	14	11	13	11	11	13	6	4
SSW	3	3	3	4	4	3	4	4	4	5	3	2
SW	5	4	4	4	4	3	4	3	5	5	5	5
WSW	6	5	4	4	4	4	3	4	5	5	6	7
W	13	13	10	11	8	7	7	8	8	8	12	17
WNW	10	9	7	7	5	4	4	4	5	6	9	10
NW	16	17	17	12	8	8	8	8	9	11	16	17
NNW	18	21	20	18	14	15	15	15	14	13	17	18

**Table 3:** Monthly frequencies of mean wind speeds at Thala site.

Wind speed (m/s)	January	February	March	April	May	June	July	August	September	October	November	December
0	2	2	1	1	1	1	2	2	2	2	3	1
1	4	3	3	3	4	5	5	6	6	6	5	4
2	9	8	7	7	9	11	12	12	13	12	9	8
3	10	8	10	10	11	14	14	15	15	13	11	10
4	9	9	10	9	12	14	14	14	12	12	10	9
5	9	10	11	10	12	13	12	14	13	12	11	10
6	8	10	11	11	12	11	11	11	11	11	10	10
7	10	10	10	11	10	10	10	8	9	8	9	10
8	8	8	8	7	7	6	6	6	5	6	7	8
9	7	7	6	7	6	5	5	4	4	5	7	7
10	6	6	6	7	5	4	4	3	3	4	6	7
11	7	5	4	5	4	3	3	2	2	3	4	5
12	4	4	3	3	2	2	2	1	2	2	3	3
13	3	3	2	2	2	1	1	1	1	2	3	3
14	2	2	2	2	1	1	0	1	1	1	1	2
15	2	2	1	1	1	0	0	0	1	1	1	1
>16	4	4	4	3	1	1	0	0	1	1	2	4

**Table 4:** Number of sirocco days recorded in Thala site.

	January	February	March	April	May	June	July	August	September	October	November	December
Number of sirocco days	0	0	0	3	26	59	111	53	11	0	0	0

**Table 5:** Number of observations per month for the two series of measurements.

Number of observations	January	February	March	April	May	June	July	August	September	October	November	December
from1981 to 1988	111	126	148	149	173	259	385	312	319	224	116	104
At 2001	**—**	—	242	629	574	1086	1011	959	468	517	—	—

**Table 6:** Number of inversions for the computation of aerosol size distribution at Thala in 2001.

	March	April	May	June	July	August	September	October
Number of inversions	21	50	36	84	94	85	31	50
